# Supplementary material for: Soft rot pathogen Dickeya dadantii 3937 produces tailocins resembling the tails of Peduovirus P2
Source: Front Microbiol. 2023 Nov 30;14:1307349. doi: 10.3389/fmicb.2023.1307349 (PMC10719855; doi:10.3389/fmicb.2023.1307349)
Supplement: Supplementary file 1 [file Data_Sheet_1.pdf]

Supplementary Materials for

Soft rot pathogen *Dickeya dadantii* 3937 produces tailocins resembling the tails of *Peduovirus* P2

Marcin Borowicz *et al.*

Corresponding author: Robert Czajkowski. Email: robert.czajkowski@ug.edu.pl

This PDF file includes:

Figs. S1 to S4
Tables S1 to S2
References

Other Supplementary Materials for this manuscript include the following:

Data S1 to S2

Fig. S1

Host-dependent killing rate of P2D1 dickeyocins. The killing rate was calculated as the percentage (%) of the remaining viable bacterial cells of the susceptible *Dickeya* spp. strains (measured by OD_{600}) after 20 min (A) and after 120 min (B) of their incubation with P2D1 dickeyocins. The results are shown as a box plot; whiskers reflect the maximum and minimum, box sides reflect the first and third quartile and the bars reflect medians. Points indicate particular measurements (n=10). Statistically significant differences between the treatments were obtained using Welch's one-way analysis of variance followed by the Games-Howell post hoc test groups with the same letter are not significantly different (p<0.05).

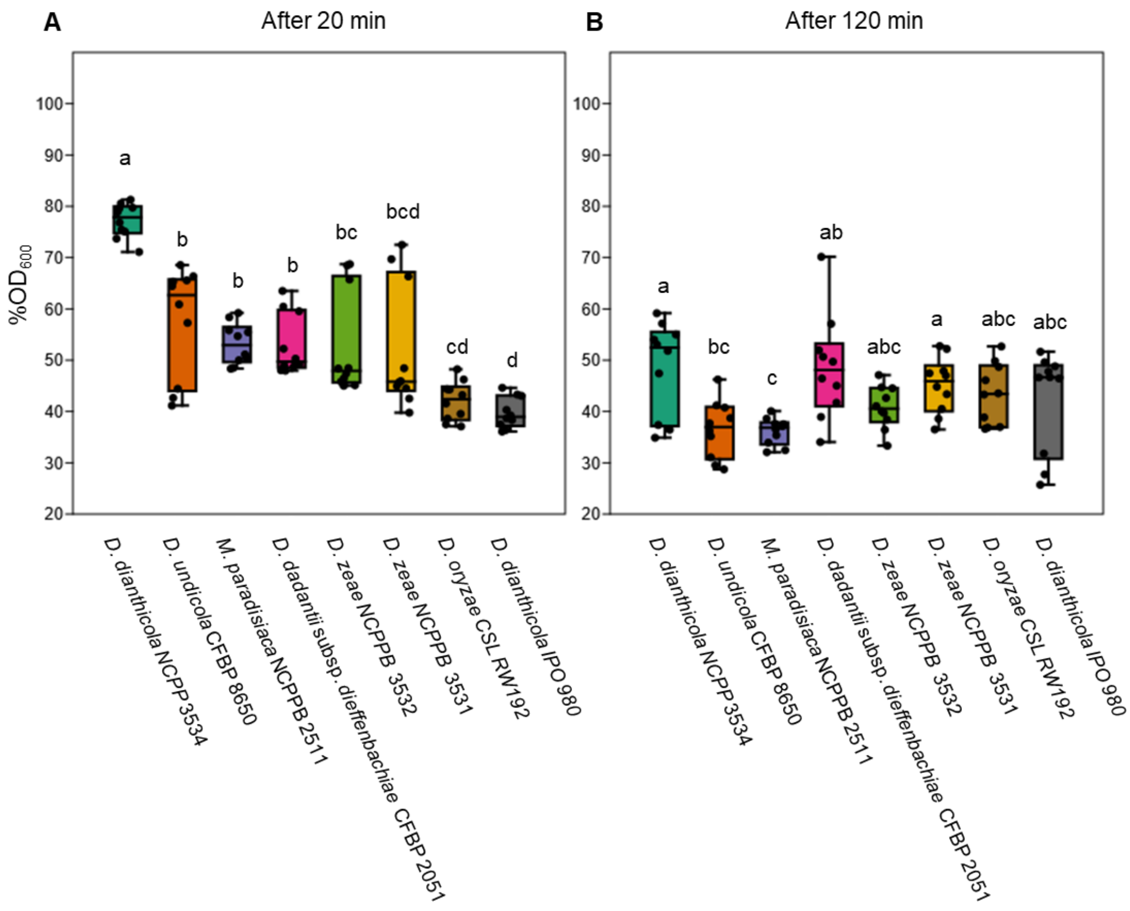

Fig. S2

Remaining activity of dickeyocins P2D1 after their binding to viable and nonviable (dead) cells of the susceptible (*M. paradisiaca* NCPPB 2511) and nonsusceptible (*D. dadantii* 3937) strains. Results are shown as box plots; the whiskers reflect the maximum and minimum, the box sides reflect the first and third quartile, and the bars reflect the medians. Points indicate particular measurements (n=9). Statistically significant differences between the treatment and the control were obtained using Kruscal-Wallis's one-way analysis of variance followed by Dunn's post hoc test. Groups with the same letter are not significantly different ($p<0.05$). AU – relative units.

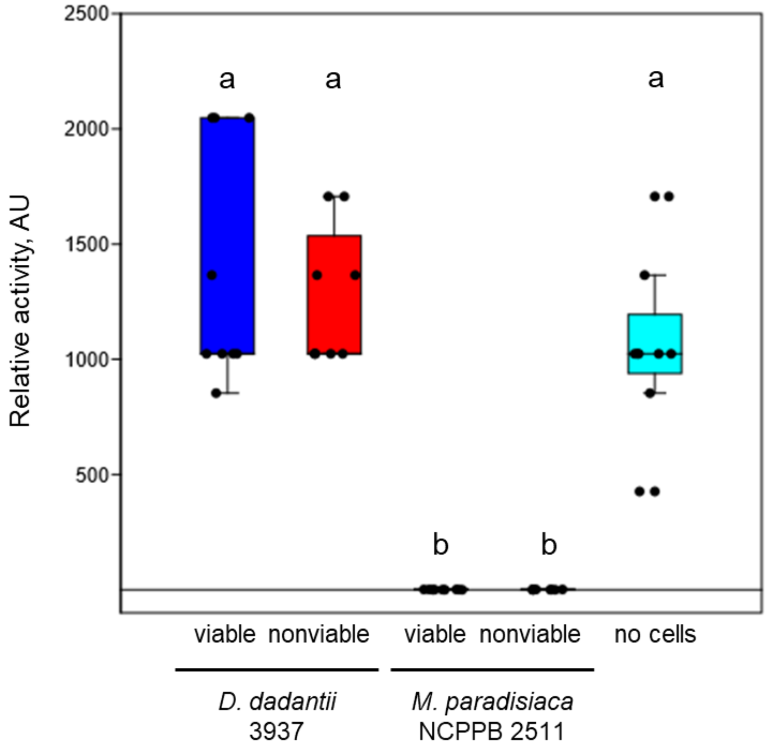

Fig. S3

Morphologic comparison of phage tail-like particles produced by T6SS defective mutant (A5587) and P2D1 dickeyocins produced by a wild type *D. dadanti* strain 3937. Representative TEM images of tailocins produced by the *Dickeya dadantii* wild-type strain and mutant A5587 (insertion in the *tssK* gene) are shown. The diameters (weight and length) were compared using TEM, with *n* representing the number of particles measured to obtain the average.

	Dimensions (nm ± SD)		Morphology in TEM	
	**Length**	**Width**		
**Wild type** *D. dadanti* **3937** *n=50*	166 ± 7	23 ± 2	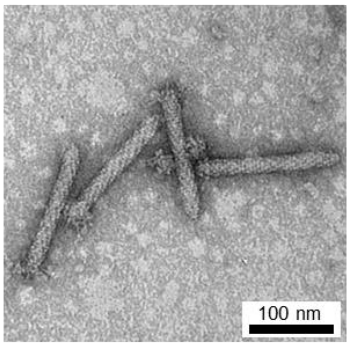	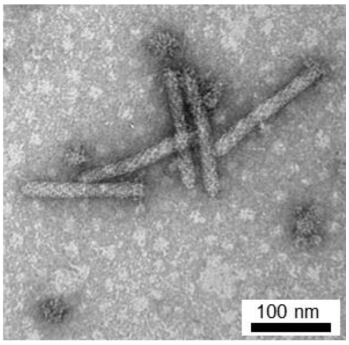
**T6SS defective mutant (A5587)** *n=25*	171 ± 10	25 ± 2	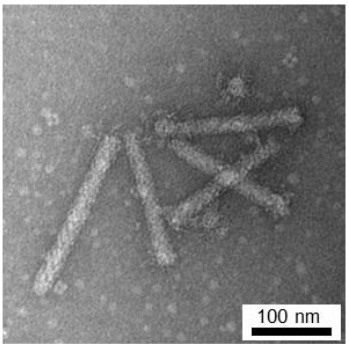	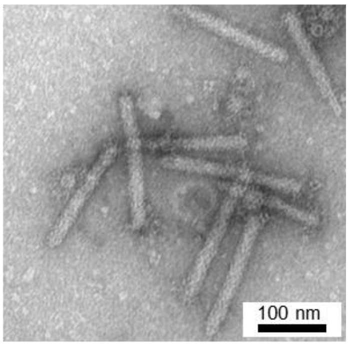

Fig. S4

Survival of *C. elegans* in the presence of P2D1 dickeyocins in the liquid killing assay. *C. elegans* growth medium supplemented with PBS without tailocins served as a control. Eight different concentrations of P2D1 were tested. Results are shown as box plots; the whiskers reflect the maximum and minimum, the box sides reflect the first and third quartile, bars reflect the medians. Points indicate particular measurements (n=9).

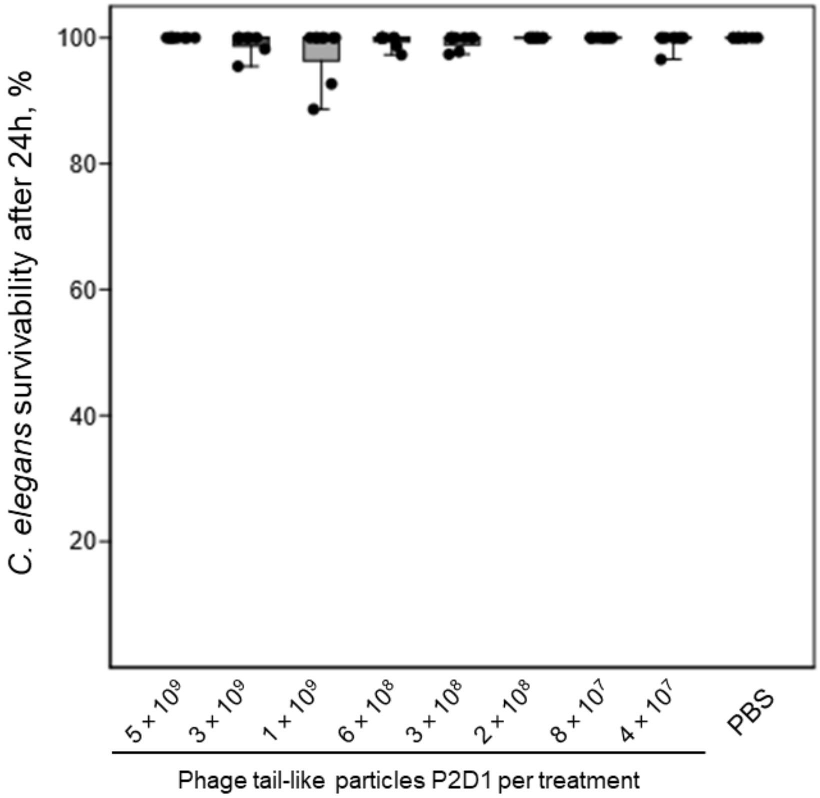

Table S1

List of strains used in this study.

Strain	Host plant/origin of isolation	Geographical origin, year of isolation	Other collection numbers	Ref.
**Soft Rot *Pectobacteriaceae***				
Dickeya chrysanthemi NCPPB 3533	*Solanum tuberosum*	United States, 1987	IFB0139	*(1)*
Dickeya chrysanthemi NCPPB 402	*Chrysanthemum morifolium*	United States, 1956*	IFB0055, CFBP 2048, ATCC 11663	*(1, 2)*
Dickeya chrysanthemi NCPPB 516	*Parthenium argentatum*	Denmark, 1957*	IFB0724, CFBP 1270	*(1)*
Dickeya dadantii 3937	*Saintpaulia sp.*	France, 1972	IFB0016	*(3)*
Dickeya dadantii DSM 18020	*Pelargonium capitatum*	Comoros, 1961*	IFB0010, CFBP 1269, NCPPB 898, SCRI 1269	*(2)*
Dickeya dadantii NCPPB 3537	*Solanum tuberosum*	Peru, 1987	IFB0127	*(1)*
Dickeya dadantii subsp. *dieffenbachiae* NCPPB 2976	*Dieffenbachia*	United States, 1977*	IFB0718, CFBP 2051	*(1, 2)*
Dickeya dianthicola IPO 980	*Solanum tuberosum*	Netherlands	IFB0140	*(4)*
Dickeya dianthicola NCPPB 3534	*Solanum tuberosum*	Netherlands, 1987	IFB0126	*(4)*
Dickeya fangzhongdai DSM 101947	*pear tree (bleeding cancer)*	China, 2009	IFB0716, CFBP 8607	*(5)*
Dickeya lacustris CFBP 8647	*water*	France, 2017	IFB0715, LMG 30899	*(6)*
Dickeya oryzae CSL RW 192	*river water*	England	IFB0220	*(1)*
Dickeya poaceiphila NCPPB 569	*Saccharum officinarum*	Australia, 1958	IFB0717, CFBP 8731	*(7)*
Dickeya solani D s0432-1	*Solanum tuberosum*	Finland, 2004	IFB0135, IPO 3295, LMG 27551	*(8)*
Dickeya solani GBBC 2040	*Solanum tuberosum*	Belgium, 2007*	IFB0484, LMG 25865	*(4)*
Dickeya solani MK10	*Solanum tuberosum*	Israel	IFB0723	*(4)*
Dickeya solani MK16	*river water*	United Kingdom	IFB0272, IPO 3494	*(1, 4)*
Dickeya sp. CSL RW 240	*river water*	England	IFB0721	*(1)*
Dickeya sp. MK7	*river water*	Scotland	IFB0275	*(1)*
Dickeya sp. NCPPB 3274	*Aglaonema*	St. Lucia, 1983	IFB0722	*(1)*
Dickeya undicola CFBP 8650	*water*	Malaysia, 2014	IFB0714, LMG 30903	*(9)*
Dickeya zeae MK19	*river water*	Scotland	IFB0719	*(1)*
Dickeya zeae NCPPB 3531	*Solanum tuberosum*	Australia, 1987*	IFB0138	*(1)*
Dickeya zeae NCPPB 3532	*Solanum tuberosum*	Australia, 1987*	IFB0720	*(1)*

Musicola paradisiaca NCPPB 2511	*Musa paradisiaca*	Colombia, 1973*	IFB0117, ATCC 33242, LMG 2542	*(1, 10)*
Pectobacterium actinidiae LMG 26003	*Actinidia chinensis*	Korea	IFB5641	*(11)*
Pectobacterium aroidearum NCPPB 929	*Zantedeschia aethiopica*	South Africa, 1959	IFB5514, LMG 2417	*(12)*
Pectobacterium atrosepticum NCPPB 549	*Solanum tuberosum*	United Kingdom, 1957	IFB5399, CFBP1526, ATCC 33260	*(13)*
Pectobacterium atrosepticum SCRI 1043	*Solanum tuberosum*	Scotland, 1985	IFB5102	*(14)*
Pectobacterium betavasculorum CFBP 2122	*Beta vulgaris cv. Saccharata*	USA, 1971	IFB5269, CFBP 2122, NCPPB 2795	*(13)*
Pectobacterium brasiliense LMG 21371	*Solanum tuberosum*	Brazil, 1999	IFB5390, ATCC BAA-417	*(15)*
Pectobacterium cacticida CFBP 3628	*Carnegiea gigantea*	USA, 1944	IFB5644, ATCC 49481, CIP 105191	*(16)*
Pectobacterium carotovorum CFBP 2046	*Solanum tuberosum*	Denmark, 1952	IFB5263, NCPPB 312, ATCC 15713	*(16)*
Pectobacterium fontis CFBP 8629	*water*	Malaysia, 2015	IFB5645, LMG30744	*(17)*
Pectobacterium parmentieri CFBP 8475	*Solanum tuberosum*	France, 2008	IFB5648, LMG 29774	*(18)*
Pectobacterium parmentieri SCC3193	*Solanum tuberosum*	Finland, 1980s	IFB5395	*(19)*
Pectobacterium peruviense CFBP 5834	*Solanum tuberosum*	Peru, 1979	IFB5232, LMG 30269; PCM 2893; SCRI 179	*(20)*
Pectobacterium polaris NCPPB 4611	*Solanum tuberosum*	Norway, 2010	IFB5646, CFBP 8603	*(21)*
Pectobacterium polonicum DPMP 315	*vegetable field*	Poland, 2016	IFB5673, LMG 31077	*(22)*
Pectobacterium punjabense CFBP 8604	*Solanum tuberosum*	Pakistan, 2017	IFB5642, LMG30622	*(23)*
Pectobacterium versatile CFBP 6051	*Solanum tuberosum*	Netherlands, 2001	IFB5636, NCPPB 3387	*(15)*
**Other strains**				
Citrobacter freundii ATCC 8090		Unknown, 1928	NCTC 9750	*(24)*
Escherichia coli ATCC 25922	*clinical isolate*	USA, 1946	DSM 1103, NCIB 12210	*(25)*
Escherichia coli ATCC 8739	*feces*			*(26)*
Escherichia coli OP50				*(27)*
Klebsiella aerogenes ATCC 51697				*(28)*
Klebsiella quasipneumoniae ATCC 700603			K6, CCUG 45421, LMG 20218	*(29)*
Pseudomonas aeruginosa PA14	*clinical islate*		DSM 19882	*(30)*
Pseudomonas aeruginosa PAO1	*clincal isolate*	Australia, 1954	DSM 22644, ATCC 15692	*(31)*
Pseudomonas donghuensis P482	*Solanum lycopersicum*	Poland, 2012		*(32)*
Serratia marcescens ATCC 14756		USA	PCI 1107	*(33)*
Staphylococcus aureus ATCC 25923	*clinical isolate*			*(34)*

* – the year of addition to the collection
NCPPB – National Collection of Plant Pathogenic Bacteria
CFBP – French Collection for Plant Associated Bacteria
DSM – German Collection of Microorganisms and Cell Cultures GmbH
ATCC – American Type Culture Collection
LMG (BCCM) – Belgian Coordinated Collections of Micro-organisms

Table S2

Determination of the concentration of P2D1 dickeyocins induced from *D. dadantii* strain 3937. To determine the concentration of the dickeyocins, three independent methods were used: (i) semiquantitative estimation by a spot test, (ii) Poisson distribution killing method, and (iii) direct particle count with NanoSight NS300. For the estimation of the number of dickeyocins, three independent inductions of P2D1 dickeyocins (=3 biological replicates) were done. Likewise, the induction of phage tail-like particles from T6SS defective mutant of *D. dadantii* strain 3937 was done using the same protocol as used for P2D1 dickeyocins and described in the Materials and Methods section. The results are shown as relative units (AU) mL^{-1} ± standard deviation, killing particles mL^{-1} ± standard deviation, and particles mL^{-1} ± standard error, respectively.

Sample		Concentration determination method		
		**Semiquantitative estimation** (spot test, AU mL^{-1} ± standard deviation)	**Poisson distribution killing method,** (killing particles mL^{-1} ± standard deviation)	**Direct particle count with NanoSight NS300**, (particles mL^{-1} ± standard error)
Inductions from strain 3937	I	2.2±1.0 × $\mathbf{10^{6}}$	1.6±1.3 × $\mathbf{10^{11}}$	2.8±0.6 × $\mathbf{10^{10}}$
	II	7.2±5.1 × $\mathbf{10^{6}}$	2.7±1.9 × $\mathbf{10^{11}}$	1.5±0.2 × $\mathbf{10^{11}}$
	III	4.8±1.7 × $\mathbf{10^{6}}$	2.9±1.7 × $\mathbf{10^{11}}$	8.8±0.5 × $\mathbf{10^{10}}$
Induction from the T6SS defective 3937 mutant		3.8±0.8 × $\mathbf{10^{6}}$	2.1±1.4 × $\mathbf{10^{11}}$	8.2±1.0 × $\mathbf{10^{10}}$
PEG purification followed by ultracentrifugation		6.1±2.2 × $\mathbf{10^{6}}$	1.2±1.1 × $\mathbf{10^{12}}$	1.0±0.1 × $\mathbf{10^{12}}$

Data S1 (separate file)
NCBI database searches using blastp and blastn.

Data S2 (separate file)
Comparison of sequence homology between proteins of P2D1 and phage P2.

References

1. L. Pritchard *et al.*, Draft genome sequences of 17 isolates of the plant pathogenic bacterium dickeya. *Genome Announc* **1**, (2013).
2. R. Samson *et al.*, Transfer of Pectobacterium chrysanthemi (Burkholder et al. 1953) Brenner et al. 1973 and Brenneria paradisiaca to the genus Dickeya gen. nov. as Dickeya chrysanthemi comb. nov. and Dickeya paradisiaca comb. nov. and delineation of four novel species, Dickeya dadantii sp. nov., Dickeya dianthicola sp. nov., Dickeya dieffenbachiae sp. nov. and Dickeya zeae sp. nov. *Int J Syst Evol Microbiol* **55**, 1415-1427 (2005).
3. A. Kotoujansky, M. Lemattre, P. Boistard, Utilization of a thermosensitive episome bearing transposon TN10 to isolate Hfr donor strains of Erwinia carotovora subsp. chrysanthemi. *J Bacteriol* **150**, 122-131 (1982).
4. L. Pritchard *et al.*, Draft Genome Sequences of Four Dickeya dianthicola and Four Dickeya solani Strains. *Genome Announc* **1**, 10.1128/genomea.00087-00012 (2013).
5. Y. Tian *et al.*, Dickeyafangzhongdai sp. nov., a plant-pathogenic bacterium isolated from pear trees (Pyrus pyrifolia). *Int J Syst Evol Microbiol* **66**, 2831-2835 (2016).
6. N. Hugouvieux-Cotte-Pattat, C. Jacot-des-Combes, J. Briolay, Dickeya lacustris sp. nov., a water-living pectinolytic bacterium isolated from lakes in France. *Int J Syst Evol Microbiol* **69**, 721-726 (2019).
7. N. Hugouvieux-Cotte-Pattat, C. Brochier-Armanet, J. P. Flandrois, S. Reverchon, Dickeya poaceiphila sp. nov., a plant-pathogenic bacterium isolated from sugar cane (Saccharum officinarum). *Int J Syst Evol Microbiol* **70**, 4508-4514 (2020).
8. S. Khayi, P. Blin, T. M. Chong, K. G. Chan, D. Faure, Complete Chromosome and Plasmid Sequences of Two Plant Pathogens, Dickeya solani Strains D s0432-1 and PPO 9019. *Genome Announc* **6**, 10.1128/genomea.00233-00218 (2018).
9. S. Oulghazi *et al.*, Dickeya undicola sp. nov., a novel species for pectinolytic isolates from surface waters in Europe and Asia. *Int J Syst Evol Microbiol* **69**, 2440-2444 (2019).
10. N. Hugouvieux-Cotte-Pattat, C. J. des-Combes, J. Briolay, L. Pritchard, Proposal for the creation of a new genus Musicola gen. nov., reclassification of Dickeya paradisiaca (Samson et al. 2005) as Musicola paradisiaca comb. nov. and description of a new species Musicola keenii sp. nov. *Int J Syst Evol Microbiol* **71**, (2021).
11. Y. J. Koh *et al.*, EMERGING THREATS TO THE KIWIFRUIT INDUSTRY Pectobacterium carotovorum subsp actinidiae subsp nov., a new bacterial pathogen causing canker-like symptoms in yellow kiwifruit, Actinidia chinensis. *New Zealand Journal of Crop and Horticultural Science* **40**, 269-279 (2012).

12. S. Nabhan, S. H. De Boer, E. Maiss, K. Wydra, Pectobacterium aroidearum sp. nov., a soft rot pathogen with preference for monocotyledonous plants. *Int J Syst Evol Microbiol* **63**, 2520-2525 (2013).
13. L. Gardan, C. Gouy, R. Christen, R. Samson, Elevation of three subspecies of Pectobacterium carotovorum to species level: Pectobacterium atrosepticum sp. nov., Pectobacterium betavasculorum sp. nov. and Pectobacterium wasabiae sp. nov. *Int J Syst Evol Microbiol* **53**, 381-391 (2003).
14. K. S. Bell *et al.*, Genome sequence of the enterobacterial phytopathogen Erwinia carotovora subsp. atroseptica and characterization of virulence factors. *Proc Natl Acad Sci U S A* **101**, 11105-11110 (2004).
15. P. Portier *et al.*, Elevation of Pectobacterium carotovorum subsp. odoriferum to species level as Pectobacterium odoriferum sp. nov., proposal of Pectobacterium brasiliense sp. nov. and Pectobacterium actinidiae sp. nov., emended description of Pectobacterium carotovorum and description of Pectobacterium versatile sp. nov., isolated from streams and symptoms on diverse plants. *Int J Syst Evol Microbiol* **69**, 3207-3216 (2019).
16. L. Hauben *et al.*, Phylogenetic position of phytopathogens within the Enterobacteriaceae. *Syst Appl Microbiol* **21**, 384-397 (1998).
17. S. Oulghazi *et al.*, Transfer of the waterfall source isolate Pectobacterium carotovorum M022 to Pectobacterium fontis sp. nov., a deep-branching species within the genus Pectobacterium. *Int J Syst Evol Microbiol* **69**, 470-475 (2019).
18. S. Khayi *et al.*, Transfer of the potato plant isolates of Pectobacterium wasabiae to Pectobacterium parmentieri sp. nov. *Int J Syst Evol Microbiol* **66**, 5379-5383 (2016).
19. J. P. Koskinen *et al.*, Genome sequence of Pectobacterium sp. strain SCC3193. *J Bacteriol* **194**, 6004 (2012).
20. M. Waleron *et al.*, Transfer of Pectobacterium carotovorum subsp. carotovorum strains isolated from potatoes grown at high altitudes to Pectobacterium peruviense sp. nov. *Syst Appl Microbiol* **41**, 85-93 (2018).
21. M. W. Dees, E. Lysoe, S. Rossmann, J. Perminow, M. B. Brurberg, Pectobacterium polaris sp. nov., isolated from potato (Solanum tuberosum). *Int J Syst Evol Microbiol* **67**, 5222-5229 (2017).
22. M. Waleron *et al.*, Pectobacterium polonicum sp. nov. isolated from vegetable fields. *Int J Syst Evol Microbiol* **69**, 1751-1759 (2019).
23. S. Sarfraz *et al.*, Pectobacterium punjabense sp. nov., isolated from blackleg symptoms of potato plants in Pakistan. *Int J Syst Evol Microbiol* **68**, 3551-3556 (2018).
24. C. H. Werkman, G. F. Gillen, Bacteria Producing Trimethylene Glycol. *J Bacteriol* **23**, 167-182 (1932).
25. T. D. Minogue *et al.*, Complete Genome Assembly of Escherichia coli ATCC 25922, a Serotype O6 Reference Strain. *Genome Announc* **2**, (2014).
26. P. Broxton, P. M. Woodcock, P. Gilbert, A study of the antibacterial activity of some polyhexamethylene biguanides towards Escherichia coli ATCC 8739. *J Appl Bacteriol* **54**, 345-353 (1983).
27. S. E. Hulme *et al.*, Lifespan-on-a-chip: microfluidic chambers for performing lifelong observation of C. elegans. *Lab Chip* **10**, 589-597 (2010).
28. B. J. Tindall, G. Sutton, G. M. Garrity, Enterobacter aerogenes Hormaeche and Edwards 1960 (Approved Lists 1980) and Klebsiella mobilis Bascomb et al. 1971 (Approved Lists 1980) share the same nomenclatural type (ATCC 13048) on the Approved Lists and are homotypic synonyms, with consequences for the name Klebsiella mobilis Bascomb et al. 1971 (Approved Lists 1980). *Int J Syst Evol Microbiol* **67**, 502-504 (2017).
29. A. G. Elliott, D. Ganesamoorthy, L. Coin, M. A. Cooper, M. D. Cao, Complete Genome Sequence of Klebsiella quasipneumoniae subsp. similipneumoniae Strain ATCC 700603. *Genome Announc* **4**, (2016).

30. H. Mikkelsen, R. McMullan, A. Filloux, The Pseudomonas aeruginosa reference strain PA14 displays increased virulence due to a mutation in ladS. *PLoS One* **6**, e29113 (2011).
31. B. W. Holloway, Genetic recombination in Pseudomonas aeruginosa. *J Gen Microbiol* **13**, 572-581 (1955).
32. D. M. Krzyzanowska *et al.*, RHIZOSPHERE BACTERIA AS POTENTIAL BIOCONTROL AGENTS AGAINST SOFT ROT CAUSED BY VARIOUS PECTOBACTERIUM AND DICKEYA spp. STRAINS. *Journal of Plant Pathology* **94**, 367-378 (2012).
33. E. Moyer, G. Cole, E. Harding, M. Jamieson-Popp, J. L. Fuls, Comparative Recovery of Serratia marcescens Using Bags versus Gloves as Described in ASTM E1174-21 Health Care Personnel Handwash Method. *Microbiol Spectr* **11**, e0128823 (2023).
34. F. Hamadi *et al.*, Effect of pH on distribution and adhesion of Staphylococcus aureus to glass. *Journal of Adhesion Science and Technology* **19**, 73-85 (2005).
